# Miscellaneous

**Published:** 1888-07

**Authors:** 


### MISCELLANEOUS.

MUCH of the so-called ivory now in use is simply potato. A good, sound potato washed in diluted sulphuric acid, then boiled in the same solution and then slowly dried, is all ready to be turned into buttons, poker chips and innumerable other things that ivory was used for once upon a time.

SUNSHINE.—After thirty years of travelling in all climates of the earth, we are satisfied that sunlight is one of the most important factors of life, and indispensable to vigorous health. Shade tends to weaken the skin, and acts deleteriously on the nerves and liver. We always, therefore, wherever we sojourn select a room on the sunny side of the house, making little account of the outlook in other respects. The finest prospect towards the north is not for a moment to weigh against the rays of the cheering, life-giving orb of day. We recommend this practice to our readers.—*Dr. Joseph Simms.*

BLOSSOMS PRODUCE AN EPIDEMIC.—"Do you know what makes May such an unhealthy month in Atlanta?" asked a citizen. "Why it is all on account of those abominable alianthus trees. In 1878 there was an ordinance to have them all cut down and allow no more to be planted, but still they flourish and bring sickness and death. May is their full blooming time, and consequently everybody is sick during that month. The flower is rank poison to children and adults having any kind of membranous trouble."—*Atlanta Constitution.*

INSTINCT OR REASON.—It has often been a puzzling question to know if birds hatch and bring up their young by instinct, or whether they employ a certain reasoning power in the construction of their nests, in the patient sitting on the eggs, in the choice of food for their young nestlings. Many anecdotes seem to prove that the generality of birds, and especially swallows, sparrows and storks employ something more than mere instinct in the conservation and rearing of their fledgling; but on the other hand, one is inclined to think that birds cannot recognize their young from the young of other birds, even of different species. A hen will bring up a whole brood of ducklings, and everyone is familiar with the desperate efforts of the foster-mother to prevent her yellow downed charges from dabbling in the horse pond; yet one would think that the differences of form in bill and feet between ducklings and chicks would be sufficient to give an inkling to the hen that she had been "imposed" upon.

The cuckoo knows very well her egg will be hatched and the young one brought up by the unfortunate bird whose nest has been turned into a foundling institution. These and other similar facts seem to prove that birds cannot distinguish their young, even from the young of dissimilar species. But a curious fact that has taken place in the colony of canaries that I have, has led me to think that some species of birds at least, better developed, can really distinguish their "babies." I had four hen canaries setting at one time; one of the nests, containing three eggs, was upset by accident and only one egg escaped "poaching." The hen bird refused to continue sitting and the next day I placed the abandoned egg in another nest where two eggs, laid at the same time, were being hatched.

The orphan bird and his adopted brothers came to the light of day in due time, and all three were taken good care of by the parent birds. Meanwhile, the first nest contained three new eggs, and I considered the canary sufficiently compensated for the loss of her first brood.

Now comes the curious part of the story. The abandoned egg had no sooner "sprouted" into an ugly little mass of open beak fleshy-colored embryo of a bird, than the real mother, abandoning her new eggs, started forth and drove the foster-mother off the nest and fed her "child" herself, disdaining the other two which did not belong to her. The fostermother made no distinction between the three, but as I saw that a war "to the beak" had been declared between the two female birds, I had to take the youngster out of his nest, replace him in his first home, where he helped his mother in hatching out the three eggs. Both birds remained quietly each in her own corner, after this.

The query now comes, was it instinct or was it reason, that enabled the canary to recognize the fledgling, which she had abandoned while in the egg, amongst the others in a strange nest.—G. D. Home, in the *R. P. Journal*.

### ABU AL ASCHAD.

Abu al Aschad, of his tribe beloved,
For deeds of mercy, by compassion moved,
All unattended on a summer's day
Rode his proud steed upon his homeward way,
Where by the roadside, waiting and alone,
A poor misshapen wretch made piteous moan.

Al Aschad paused, and drawing to his side,
Gave up his saddle that the cripple ride,
E'en as he prayed, if but a little way
Toward Merea's holy shrine that eastward lay,
When of a sudden giving spur and rein,
The favored mendicant fled o'er the plain,
A very cheat no longer travel-sore
Nor faint and crippled seeming as before,
But bold, exultant, wanting not in skill,
To guide the fleeting steed where e'er he will.

A moment stood Al Aschad, bowed of head,
Then came his speech, 'tis not my steed, he said,
Of faultless pedigree ; 'tis not my geld,
Nor yet my jewels, all so dearly held,
And robbed of me, that I do most deplore,
But 'tis the fear that in my journeyings o'er
Life's common ways, still dreading like abuse,
Some needful charity, I might refuse.

La Croix.

